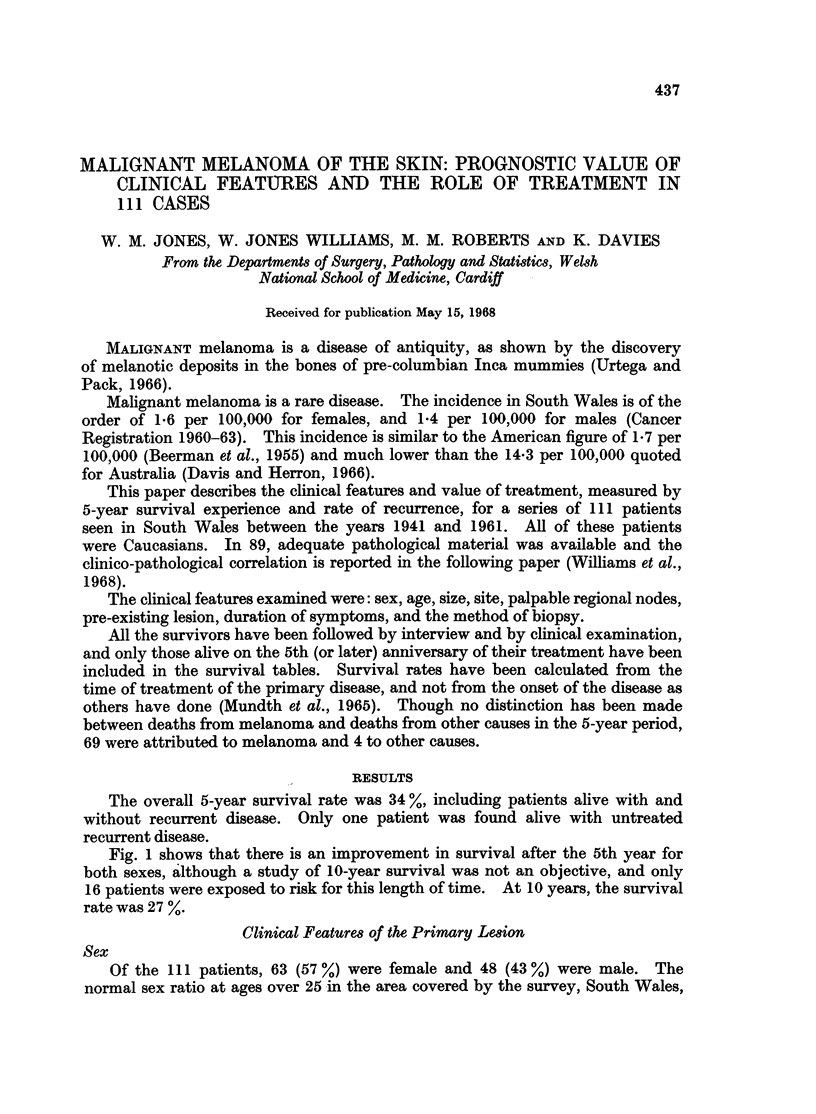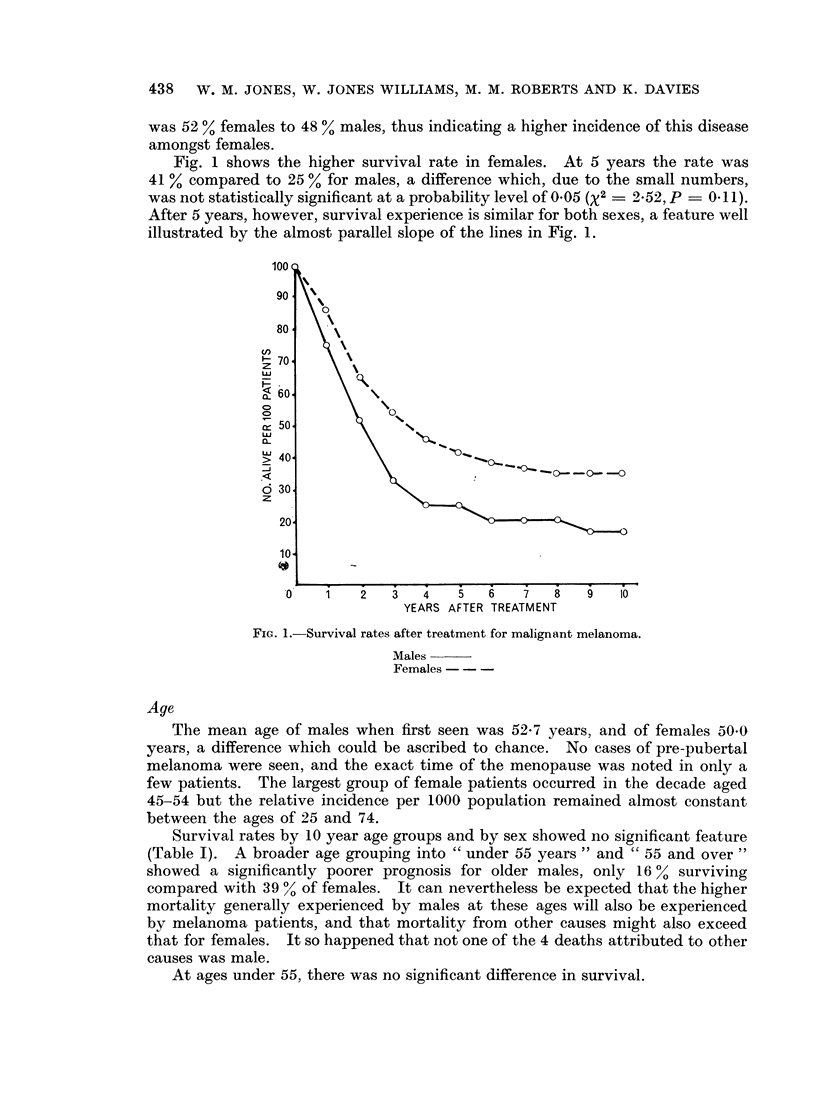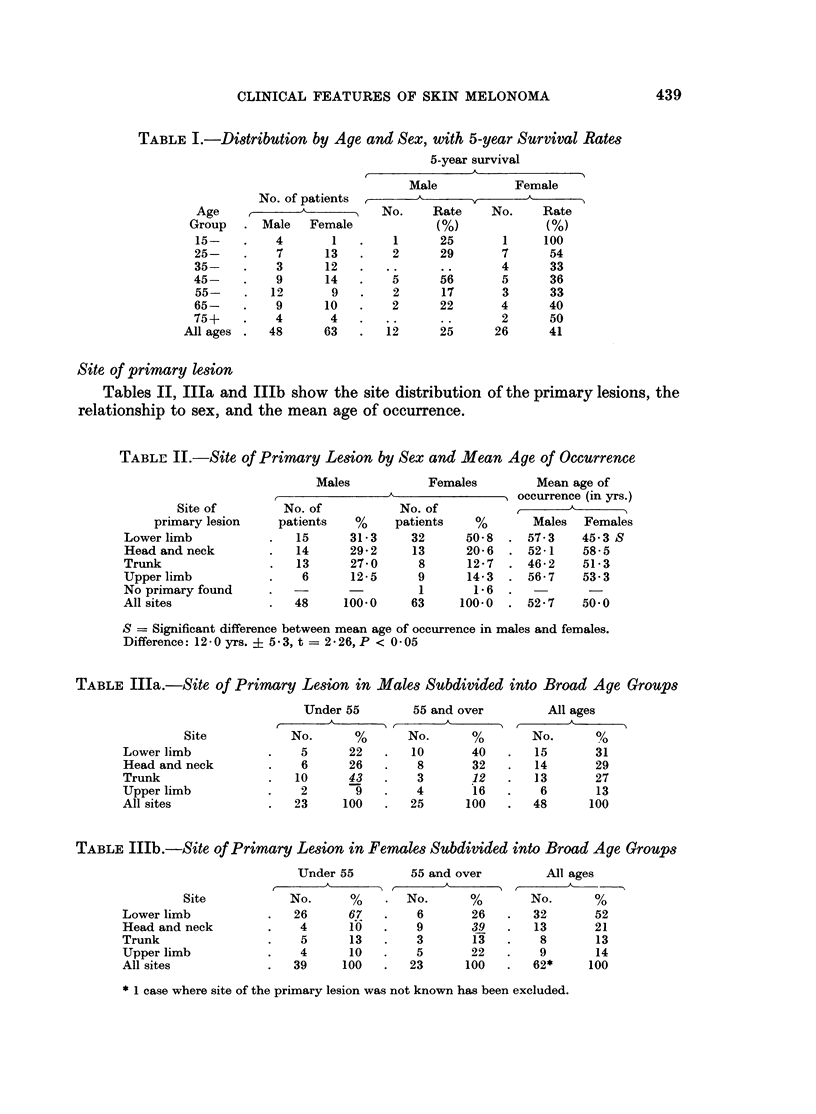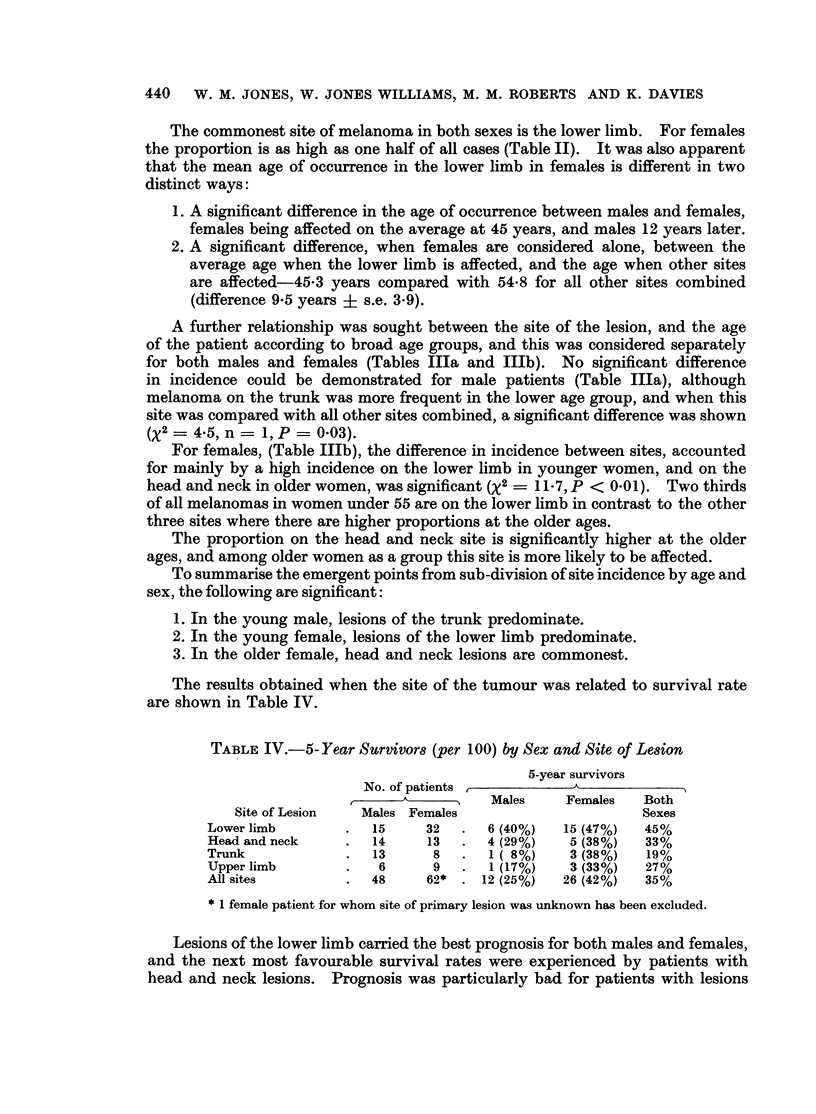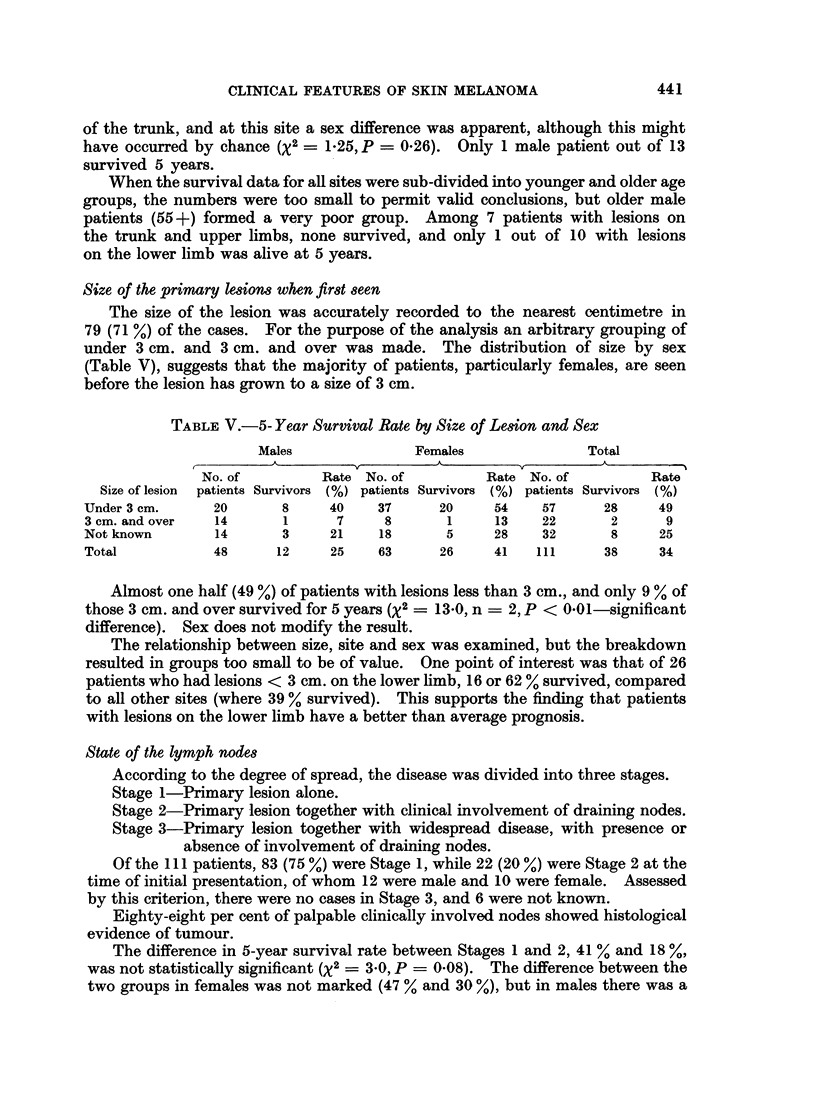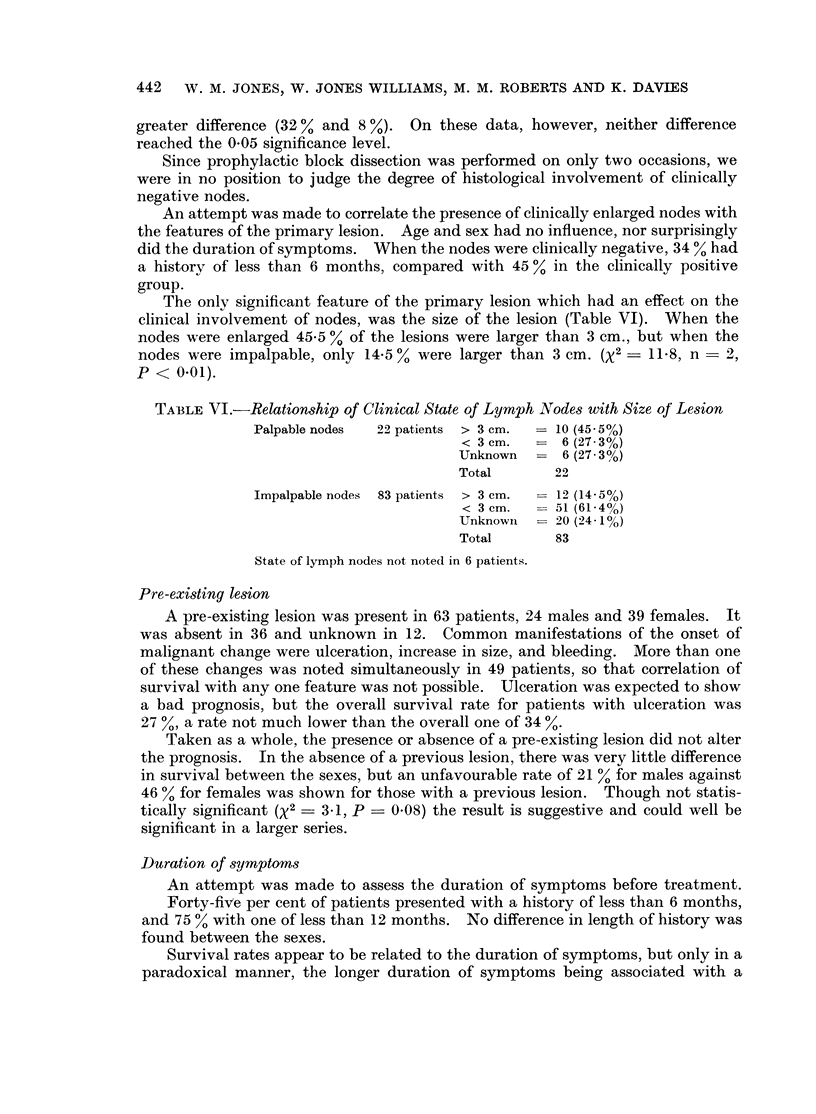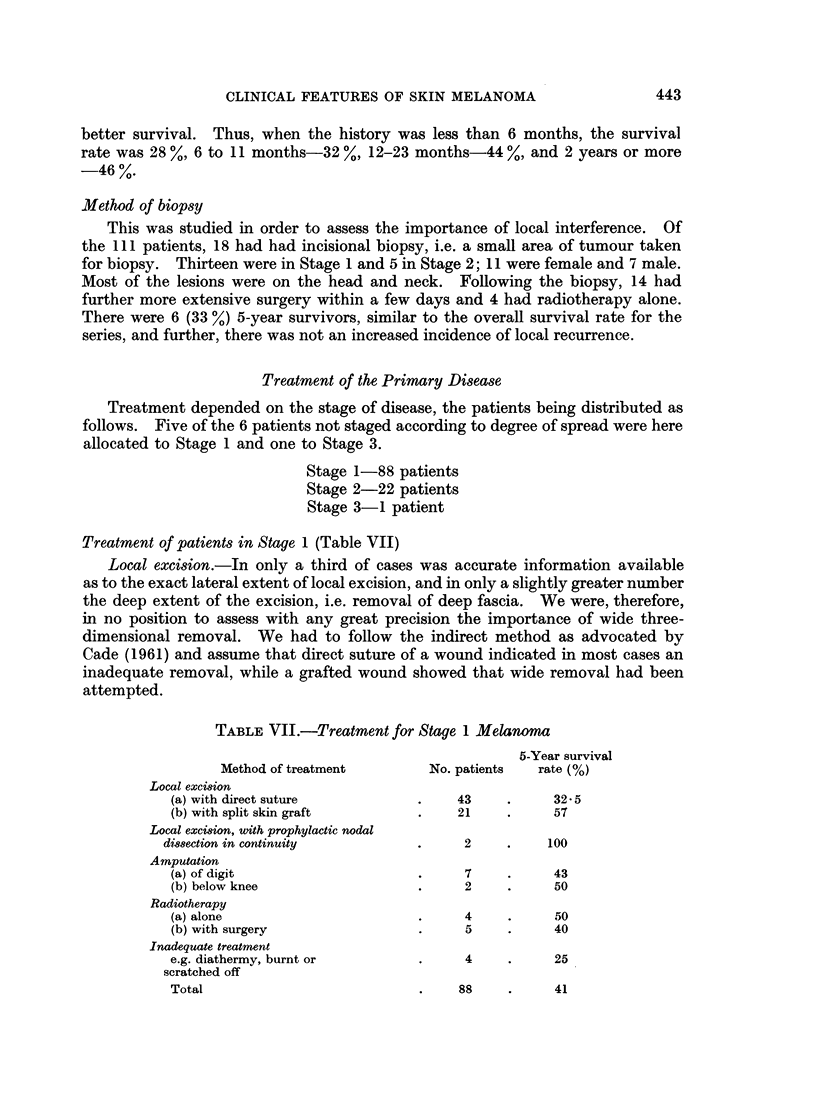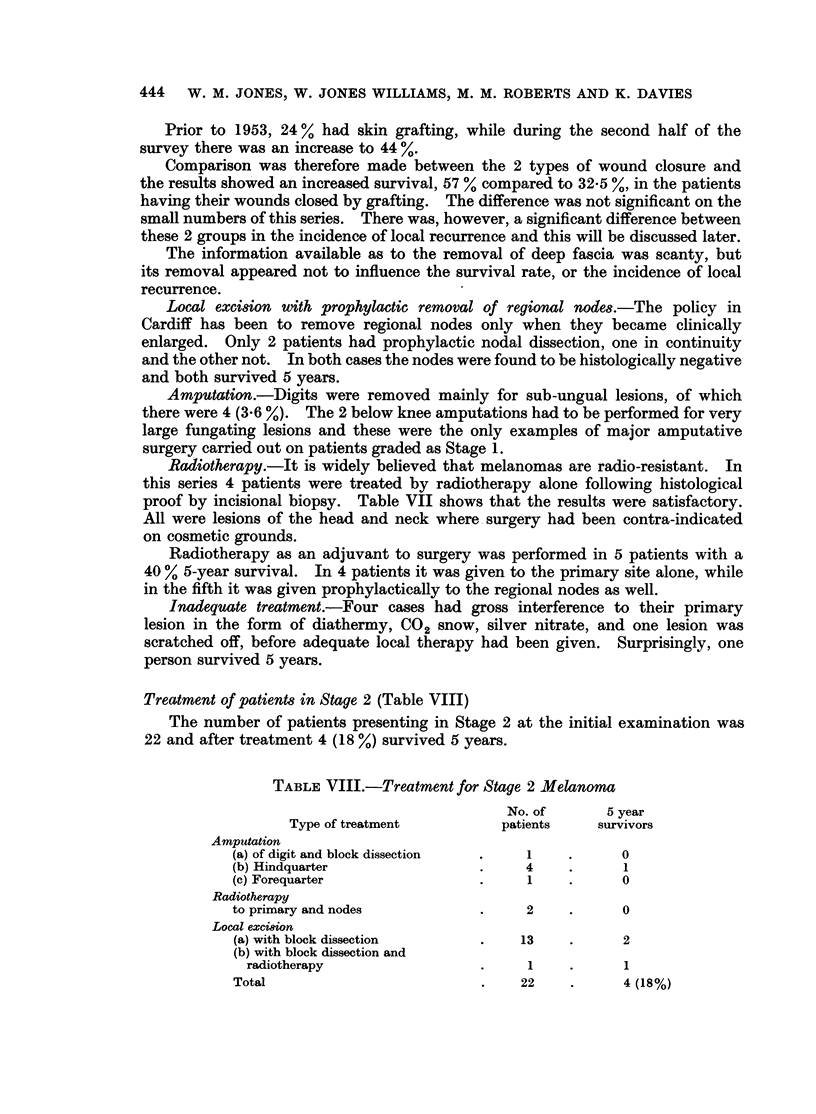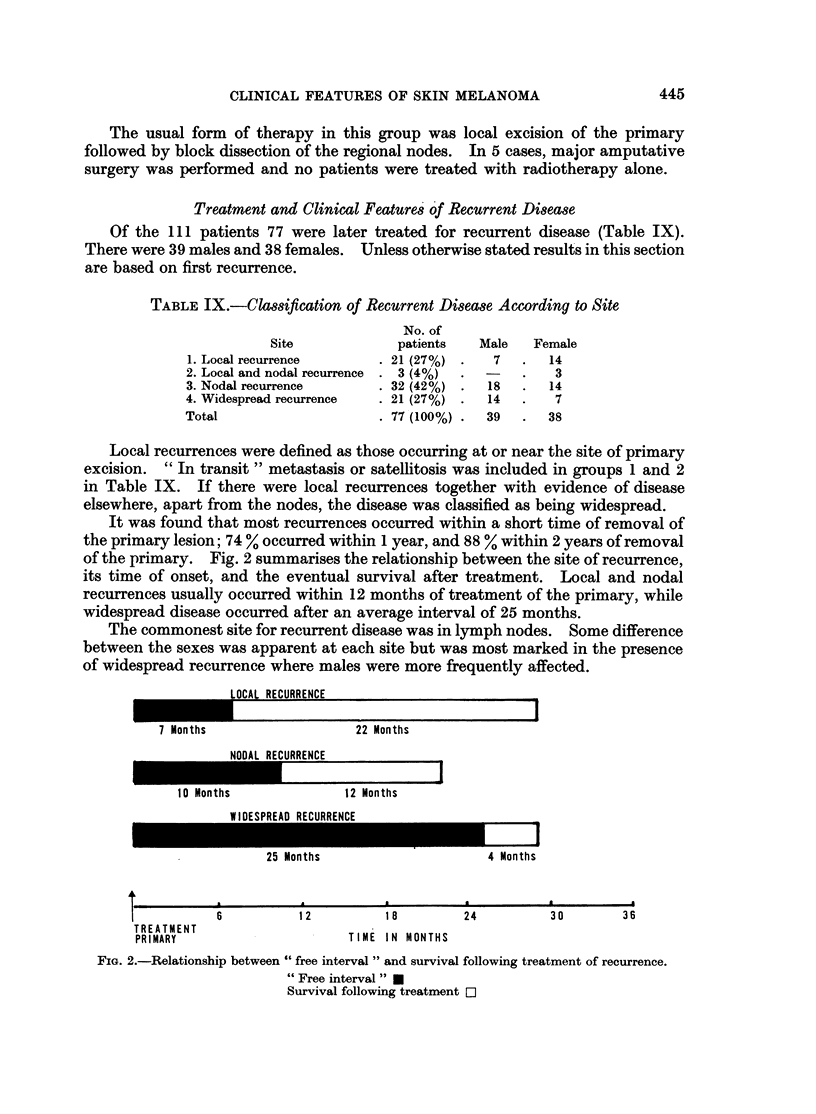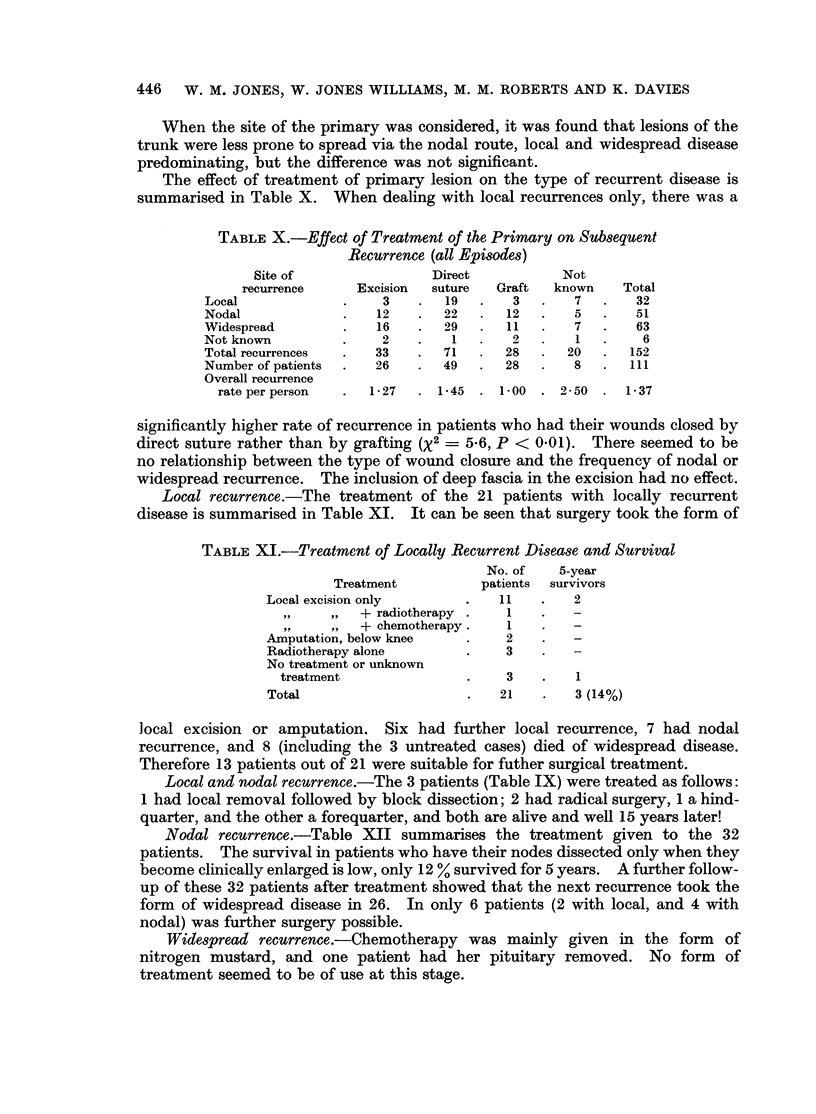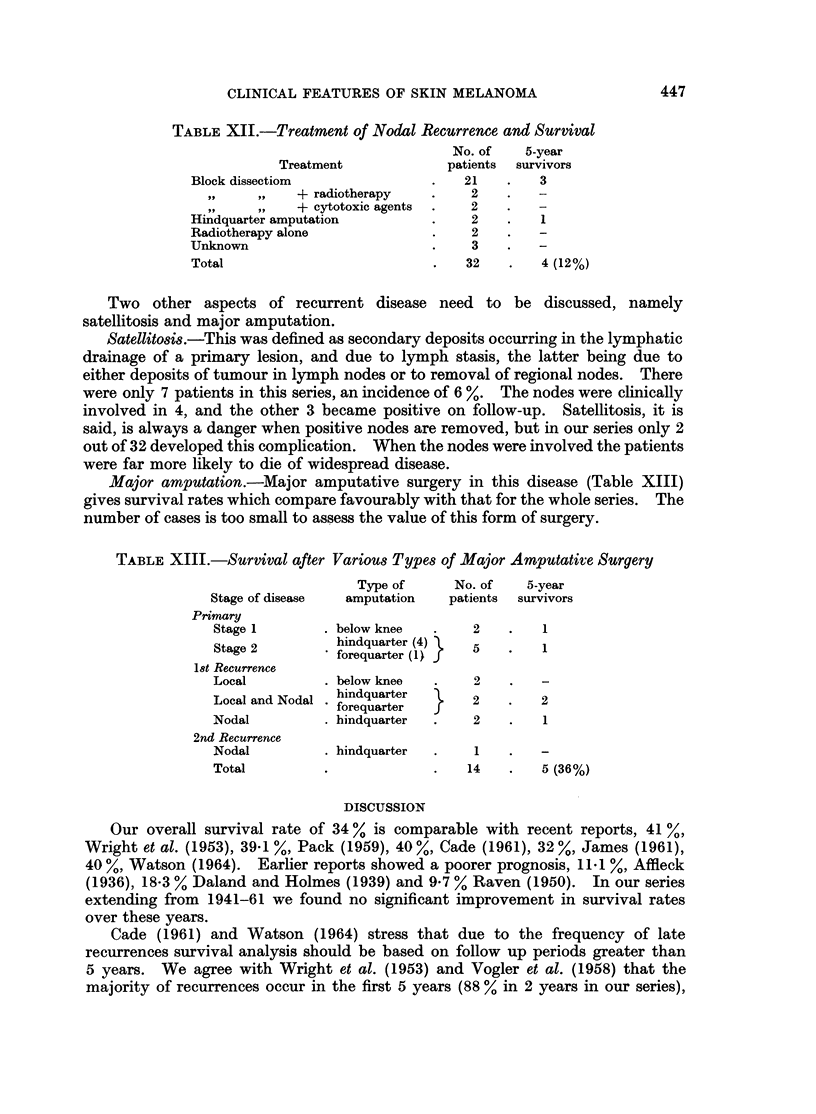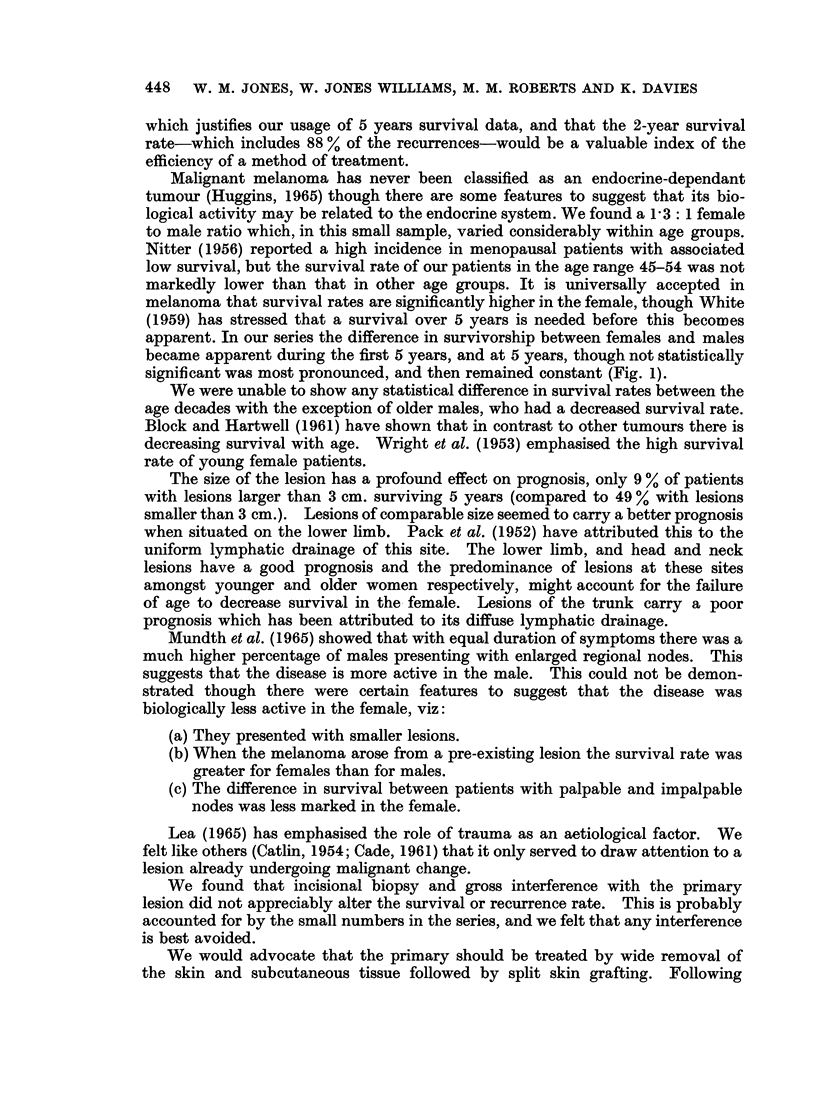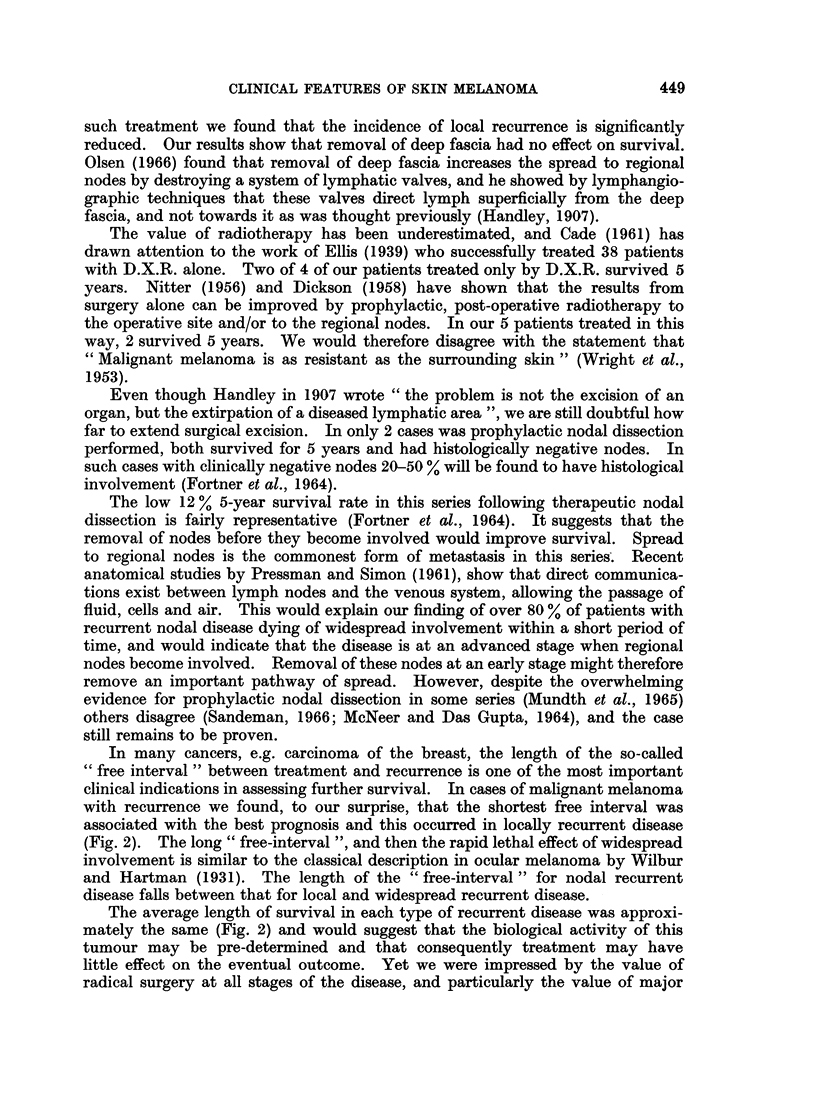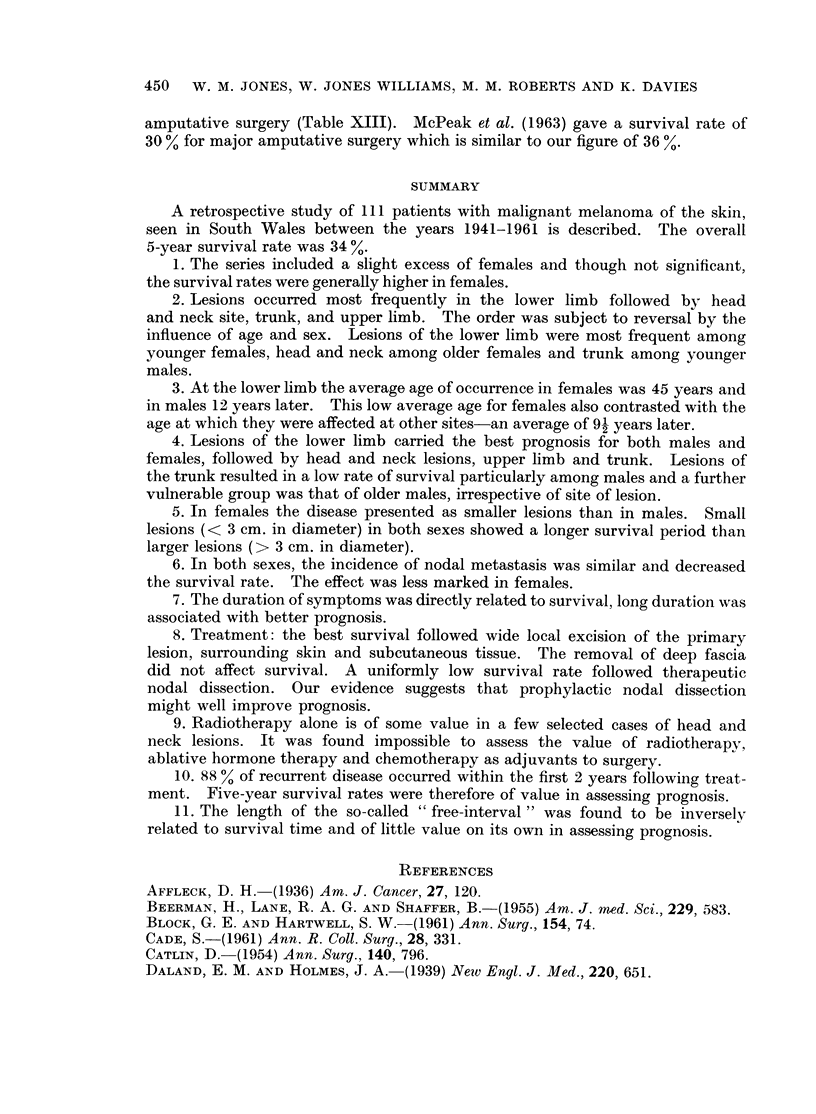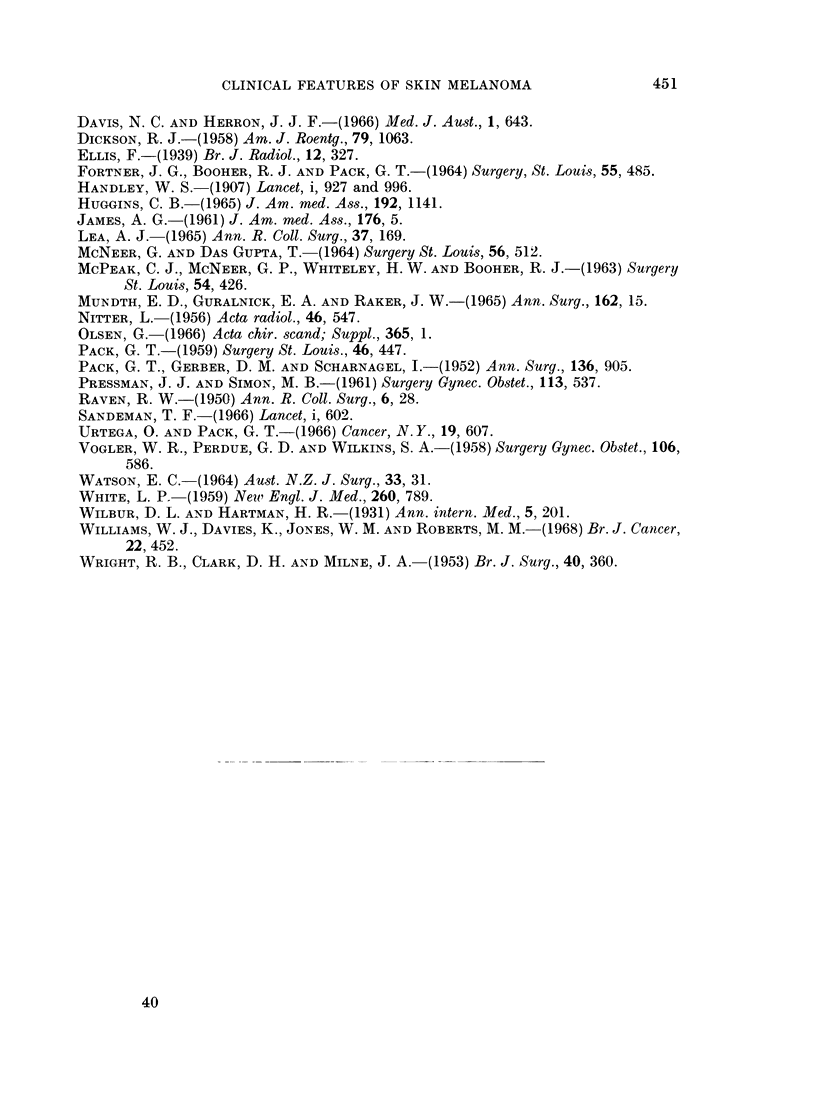# Malignant melanoma of the skin: prognostic value of clinical features and the role of treatment in 111 cases.

**DOI:** 10.1038/bjc.1968.51

**Published:** 1968-09

**Authors:** W. M. Jones, W. J. Williams, M. M. Roberts, K. Davies


### MALIGNANT MELANOMA OF THE SKIN: PROGNOSTIC VALUE OF CLINICAL FEATURES AND THE ROLE OF TREATMENT IN 111 CASES

W. M. JONES, W. JONES WILLIAMS, M. M. ROBERTS AND K. DAVIES
*From the Departments of Surgery, Pathology and Statistics, Welsh National School of Medicine, Cardiff*

Received for publication May 15, 1968

MALIGNANT melanoma is a disease of antiquity, as shown by the discovery of melanotic deposits in the bones of pre-columbian Inca mummies (Urtega and Pack, 1966).

Malignant melanoma is a rare disease. The incidence in South Wales is of the order of 1·6 per 100,000 for females, and 1·4 per 100,000 for males (Cancer Registration 1960–63). This incidence is similar to the American figure of 1·7 per 100,000 (Beerman *et al.*, 1955) and much lower than the 14·3 per 100,000 quoted for Australia (Davis and Herron, 1966).

This paper describes the clinical features and value of treatment, measured by 5-year survival experience and rate of recurrence, for a series of 111 patients seen in South Wales between the years 1941 and 1961. All of these patients were Caucasians. In 89, adequate pathological material was available and the clinico-pathological correlation is reported in the following paper (Williams *et al.*, 1968).

The clinical features examined were: sex, age, size, site, palpable regional nodes, pre-existing lesion, duration of symptoms, and the method of biopsy.

All the survivors have been followed by interview and by clinical examination, and only those alive on the 5th (or later) anniversary of their treatment have been included in the survival tables. Survival rates have been calculated from the time of treatment of the primary disease, and not from the onset of the disease as others have done (Mundth *et al.*, 1965). Though no distinction has been made between deaths from melanoma and deaths from other causes in the 5-year period, 69 were attributed to melanoma and 4 to other causes.

### RESULTS

The overall 5-year survival rate was 34 %, including patients alive with and without recurrent disease. Only one patient was found alive with untreated recurrent disease.

Fig. 1 shows that there is an improvement in survival after the 5th year for both sexes, although a study of 10-year survival was not an objective, and only 16 patients were exposed to risk for this length of time. At 10 years, the survival rate was 27 %.

### *Clinical Features of the Primary Lesion*

*Sex*

Of the 111 patients, 63 (57 %) were female and 48 (43 %) were male. The normal sex ratio at ages over 25 in the area covered by the survey, South Wales, was 52 % females to 48 % males, thus indicating a higher incidence of this disease amongst females.

Fig. 1 shows the higher survival rate in females. At 5 years the rate was 41 % compared to 25 % for males, a difference which, due to the small numbers, was not statistically significant at a probability level of 0·05 ($\chi^2 = 2{\cdot}52, P = 0{\cdot}11$). After 5 years, however, survival experience is similar for both sexes, a feature well illustrated by the almost parallel slope of the lines in Fig. 1.

FIG. 1.—Survival rates after treatment for malignant melanoma.

Males ————
Females — — —

*Age*

The mean age of males when first seen was 52·7 years, and of females 50·0 years, a difference which could be ascribed to chance. No cases of pre-pubertal melanoma were seen, and the exact time of the menopause was noted in only a few patients. The largest group of female patients occurred in the decade aged 45–54 but the relative incidence per 1000 population remained almost constant between the ages of 25 and 74.

Survival rates by 10 year age groups and by sex showed no significant feature (Table I). A broader age grouping into " under 55 years " and " 55 and over " showed a significantly poorer prognosis for older males, only 16 % surviving compared with 39 % of females. It can nevertheless be expected that the higher mortality generally experienced by males at these ages will also be experienced by melanoma patients, and that mortality from other causes might also exceed that for females. It so happened that not one of the 4 deaths attributed to other causes was male.

At ages under 55, there was no significant difference in survival.

TABLE I.—*Distribution by Age and Sex, with 5-year Survival Rates*

| Age Group | No. of patients: Male | No. of patients: Female | 5-year survival: Male No. | 5-year survival: Male Rate (%) | 5-year survival: Female No. | 5-year survival: Female Rate (%) |
|---|---|---|---|---|---|---|
| 15– | 4 | 1 | 1 | 25 | 1 | 100 |
| 25– | 7 | 13 | 2 | 29 | 7 | 54 |
| 35– | 3 | 12 | .. | .. | 4 | 33 |
| 45– | 9 | 14 | 5 | 56 | 5 | 36 |
| 55– | 12 | 9 | 2 | 17 | 3 | 33 |
| 65– | 9 | 10 | 2 | 22 | 4 | 40 |
| 75+ | 4 | 4 | .. | .. | 2 | 50 |
| All ages | 48 | 63 | 12 | 25 | 26 | 41 |

*Site of primary lesion*

Tables II, IIIa and IIIb show the site distribution of the primary lesions, the relationship to sex, and the mean age of occurrence.

TABLE II.—*Site of Primary Lesion by Sex and Mean Age of Occurrence*

| Site of primary lesion | Males: No. of patients | Males: % | Females: No. of patients | Females: % | Mean age of occurrence (in yrs.): Males | Mean age of occurrence (in yrs.): Females |
|---|---|---|---|---|---|---|
| Lower limb | 15 | 31·3 | 32 | 50·8 | 57·3 | 45·3 *S* |
| Head and neck | 14 | 29·2 | 13 | 20·6 | 52·1 | 58·5 |
| Trunk | 13 | 27·0 | 8 | 12·7 | 46·2 | 51·3 |
| Upper limb | 6 | 12·5 | 9 | 14·3 | 56·7 | 53·3 |
| No primary found | — | — | 1 | 1·6 | — | — |
| All sites | 48 | 100·0 | 63 | 100·0 | 52·7 | 50·0 |

*S* = Significant difference between mean age of occurrence in males and females.
Difference: 12·0 yrs. ± 5·3, t = 2·26, $P < 0·05$

TABLE IIIa.—*Site of Primary Lesion in Males Subdivided into Broad Age Groups*

| Site | Under 55: No. | Under 55: % | 55 and over: No. | 55 and over: % | All ages: No. | All ages: % |
|---|---|---|---|---|---|---|
| Lower limb | 5 | 22 | 10 | 40 | 15 | 31 |
| Head and neck | 6 | 26 | 8 | 32 | 14 | 29 |
| Trunk | 10 | *43* | 3 | *12* | 13 | 27 |
| Upper limb | 2 | 9 | 4 | 16 | 6 | 13 |
| All sites | 23 | 100 | 25 | 100 | 48 | 100 |

TABLE IIIb.—*Site of Primary Lesion in Females Subdivided into Broad Age Groups*

| Site | Under 55: No. | Under 55: % | 55 and over: No. | 55 and over: % | All ages: No. | All ages: % |
|---|---|---|---|---|---|---|
| Lower limb | 26 | *67* | 6 | 26 | 32 | 52 |
| Head and neck | 4 | 10 | 9 | *39* | 13 | 21 |
| Trunk | 5 | 13 | 3 | 13 | 8 | 13 |
| Upper limb | 4 | 10 | 5 | 22 | 9 | 14 |
| All sites | 39 | 100 | 23 | 100 | 62* | 100 |

* 1 case where site of the primary lesion was not known has been excluded.

The commonest site of melanoma in both sexes is the lower limb. For females the proportion is as high as one half of all cases (Table II). It was also apparent that the mean age of occurrence in the lower limb in females is different in two distinct ways:

1. A significant difference in the age of occurrence between males and females, females being affected on the average at 45 years, and males 12 years later.
2. A significant difference, when females are considered alone, between the average age when the lower limb is affected, and the age when other sites are affected—45·3 years compared with 54·8 for all other sites combined (difference 9·5 years $\pm$ s.e. 3·9).

A further relationship was sought between the site of the lesion, and the age of the patient according to broad age groups, and this was considered separately for both males and females (Tables IIIa and IIIb). No significant difference in incidence could be demonstrated for male patients (Table IIIa), although melanoma on the trunk was more frequent in the lower age group, and when this site was compared with all other sites combined, a significant difference was shown ($\chi^2 = 4{\cdot}5$, n $= 1, P = 0{\cdot}03$).

For females, (Table IIIb), the difference in incidence between sites, accounted for mainly by a high incidence on the lower limb in younger women, and on the head and neck in older women, was significant ($\chi^2 = 11{\cdot}7, P < 0{\cdot}01$). Two thirds of all melanomas in women under 55 are on the lower limb in contrast to the other three sites where there are higher proportions at the older ages.

The proportion on the head and neck site is significantly higher at the older ages, and among older women as a group this site is more likely to be affected.

To summarise the emergent points from sub-division of site incidence by age and sex, the following are significant:

1. In the young male, lesions of the trunk predominate.
2. In the young female, lesions of the lower limb predominate.
3. In the older female, head and neck lesions are commonest.

The results obtained when the site of the tumour was related to survival rate are shown in Table IV.

TABLE IV.—*5-Year Survivors (per 100) by Sex and Site of Lesion*

| Site of Lesion | No. of patients: Males | No. of patients: Females | 5-year survivors: Males | 5-year survivors: Females | 5-year survivors: Both Sexes |
|---|---|---|---|---|---|
| Lower limb | 15 | 32 | 6 (40%) | 15 (47%) | 45% |
| Head and neck | 14 | 13 | 4 (29%) | 5 (38%) | 33% |
| Trunk | 13 | 8 | 1 ( 8%) | 3 (38%) | 19% |
| Upper limb | 6 | 9 | 1 (17%) | 3 (33%) | 27% |
| All sites | 48 | 62* | 12 (25%) | 26 (42%) | 35% |

* 1 female patient for whom site of primary lesion was unknown has been excluded.

Lesions of the lower limb carried the best prognosis for both males and females, and the next most favourable survival rates were experienced by patients with head and neck lesions. Prognosis was particularly bad for patients with lesions of the trunk, and at this site a sex difference was apparent, although this might have occurred by chance ($\chi^2 = 1{\cdot}25, P = 0{\cdot}26$). Only 1 male patient out of 13 survived 5 years.

When the survival data for all sites were sub-divided into younger and older age groups, the numbers were too small to permit valid conclusions, but older male patients (55+) formed a very poor group. Among 7 patients with lesions on the trunk and upper limbs, none survived, and only 1 out of 10 with lesions on the lower limb was alive at 5 years.

*Size of the primary lesions when first seen*

The size of the lesion was accurately recorded to the nearest centimetre in 79 (71 %) of the cases. For the purpose of the analysis an arbitrary grouping of under 3 cm. and 3 cm. and over was made. The distribution of size by sex (Table V), suggests that the majority of patients, particularly females, are seen before the lesion has grown to a size of 3 cm.

TABLE V.—*5-Year Survival Rate by Size of Lesion and Sex*

| | Males | | | Females | | | Total | | |
|---|---|---|---|---|---|---|---|---|---|
| Size of lesion | No. of patients | Survivors | Rate (%) | No. of patients | Survivors | Rate (%) | No. of patients | Survivors | Rate (%) |
| Under 3 cm. | 20 | 8 | 40 | 37 | 20 | 54 | 57 | 28 | 49 |
| 3 cm. and over | 14 | 1 | 7 | 8 | 1 | 13 | 22 | 2 | 9 |
| Not known | 14 | 3 | 21 | 18 | 5 | 28 | 32 | 8 | 25 |
| Total | 48 | 12 | 25 | 63 | 26 | 41 | 111 | 38 | 34 |

Almost one half (49 %) of patients with lesions less than 3 cm., and only 9 % of those 3 cm. and over survived for 5 years ($\chi^2 = 13{\cdot}0$, n $= 2, P < 0{\cdot}01$—significant difference). Sex does not modify the result.

The relationship between size, site and sex was examined, but the breakdown resulted in groups too small to be of value. One point of interest was that of 26 patients who had lesions < 3 cm. on the lower limb, 16 or 62 % survived, compared to all other sites (where 39 % survived). This supports the finding that patients with lesions on the lower limb have a better than average prognosis.

*State of the lymph nodes*

According to the degree of spread, the disease was divided into three stages.

Stage 1—Primary lesion alone.

Stage 2—Primary lesion together with clinical involvement of draining nodes.

Stage 3—Primary lesion together with widespread disease, with presence or absence of involvement of draining nodes.

Of the 111 patients, 83 (75 %) were Stage 1, while 22 (20 %) were Stage 2 at the time of initial presentation, of whom 12 were male and 10 were female. Assessed by this criterion, there were no cases in Stage 3, and 6 were not known.

Eighty-eight per cent of palpable clinically involved nodes showed histological evidence of tumour.

The difference in 5-year survival rate between Stages 1 and 2, 41 % and 18 %, was not statistically significant ($\chi^2 = 3{\cdot}0, P = 0{\cdot}08$). The difference between the two groups in females was not marked (47 % and 30 %), but in males there was a greater difference (32 % and 8 %). On these data, however, neither difference reached the 0·05 significance level.

Since prophylactic block dissection was performed on only two occasions, we were in no position to judge the degree of histological involvement of clinically negative nodes.

An attempt was made to correlate the presence of clinically enlarged nodes with the features of the primary lesion. Age and sex had no influence, nor surprisingly did the duration of symptoms. When the nodes were clinically negative, 34 % had a history of less than 6 months, compared with 45 % in the clinically positive group.

The only significant feature of the primary lesion which had an effect on the clinical involvement of nodes, was the size of the lesion (Table VI). When the nodes were enlarged 45·5 % of the lesions were larger than 3 cm., but when the nodes were impalpable, only 14·5 % were larger than 3 cm. ($\chi^2 = 11 \cdot 8$, n = 2, $P < 0 \cdot 01$).

TABLE VI.—*Relationship of Clinical State of Lymph Nodes with Size of Lesion*

| | | | |
|---|---|---|---|
| Palpable nodes | 22 patients | > 3 cm. | = 10 (45·5%) |
| | | < 3 cm. | = 6 (27·3%) |
| | | Unknown | = 6 (27·3%) |
| | | Total | 22 |
| Impalpable nodes | 83 patients | > 3 cm. | = 12 (14·5%) |
| | | < 3 cm. | = 51 (61·4%) |
| | | Unknown | = 20 (24·1%) |
| | | Total | 83 |

State of lymph nodes not noted in 6 patients.

*Pre-existing lesion*

A pre-existing lesion was present in 63 patients, 24 males and 39 females. It was absent in 36 and unknown in 12. Common manifestations of the onset of malignant change were ulceration, increase in size, and bleeding. More than one of these changes was noted simultaneously in 49 patients, so that correlation of survival with any one feature was not possible. Ulceration was expected to show a bad prognosis, but the overall survival rate for patients with ulceration was 27 %, a rate not much lower than the overall one of 34 %.

Taken as a whole, the presence or absence of a pre-existing lesion did not alter the prognosis. In the absence of a previous lesion, there was very little difference in survival between the sexes, but an unfavourable rate of 21 % for males against 46 % for females was shown for those with a previous lesion. Though not statistically significant ($\chi^2 = 3 \cdot 1$, $P = 0 \cdot 08$) the result is suggestive and could well be significant in a larger series.

*Duration of symptoms*

An attempt was made to assess the duration of symptoms before treatment.

Forty-five per cent of patients presented with a history of less than 6 months, and 75 % with one of less than 12 months. No difference in length of history was found between the sexes.

Survival rates appear to be related to the duration of symptoms, but only in a paradoxical manner, the longer duration of symptoms being associated with a better survival. Thus, when the history was less than 6 months, the survival rate was 28%, 6 to 11 months—32%, 12–23 months—44%, and 2 years or more —46%.

*Method of biopsy*

This was studied in order to assess the importance of local interference. Of the 111 patients, 18 had had incisional biopsy, i.e. a small area of tumour taken for biopsy. Thirteen were in Stage 1 and 5 in Stage 2; 11 were female and 7 male. Most of the lesions were on the head and neck. Following the biopsy, 14 had further more extensive surgery within a few days and 4 had radiotherapy alone. There were 6 (33%) 5-year survivors, similar to the overall survival rate for the series, and further, there was not an increased incidence of local recurrence.

### *Treatment of the Primary Disease*

Treatment depended on the stage of disease, the patients being distributed as follows. Five of the 6 patients not staged according to degree of spread were here allocated to Stage 1 and one to Stage 3.

Stage 1—88 patients
Stage 2—22 patients
Stage 3—1 patient

*Treatment of patients in Stage* 1 (Table VII)

*Local excision.*—In only a third of cases was accurate information available as to the exact lateral extent of local excision, and in only a slightly greater number the deep extent of the excision, i.e. removal of deep fascia. We were, therefore, in no position to assess with any great precision the importance of wide three-dimensional removal. We had to follow the indirect method as advocated by Cade (1961) and assume that direct suture of a wound indicated in most cases an inadequate removal, while a grafted wound showed that wide removal had been attempted.

TABLE VII.—*Treatment for Stage* 1 *Melanoma*

| Method of treatment | No. patients | 5-Year survival rate (%) |
|---|---|---|
| *Local excision* | | |
| (a) with direct suture | 43 | 32·5 |
| (b) with split skin graft | 21 | 57 |
| *Local excision, with prophylactic nodal dissection in continuity* | 2 | 100 |
| *Amputation* | | |
| (a) of digit | 7 | 43 |
| (b) below knee | 2 | 50 |
| *Radiotherapy* | | |
| (a) alone | 4 | 50 |
| (b) with surgery | 5 | 40 |
| *Inadequate treatment* | | |
| e.g. diathermy, burnt or scratched off | 4 | 25 |
| Total | 88 | 41 |

Prior to 1953, 24% had skin grafting, while during the second half of the survey there was an increase to 44%.

Comparison was therefore made between the 2 types of wound closure and the results showed an increased survival, 57% compared to 32·5%, in the patients having their wounds closed by grafting. The difference was not significant on the small numbers of this series. There was, however, a significant difference between these 2 groups in the incidence of local recurrence and this will be discussed later.

The information available as to the removal of deep fascia was scanty, but its removal appeared not to influence the survival rate, or the incidence of local recurrence.

*Local excision with prophylactic removal of regional nodes.*—The policy in Cardiff has been to remove regional nodes only when they became clinically enlarged. Only 2 patients had prophylactic nodal dissection, one in continuity and the other not. In both cases the nodes were found to be histologically negative and both survived 5 years.

*Amputation.*—Digits were removed mainly for sub-ungual lesions, of which there were 4 (3·6%). The 2 below knee amputations had to be performed for very large fungating lesions and these were the only examples of major amputative surgery carried out on patients graded as Stage 1.

*Radiotherapy.*—It is widely believed that melanomas are radio-resistant. In this series 4 patients were treated by radiotherapy alone following histological proof by incisional biopsy. Table VII shows that the results were satisfactory. All were lesions of the head and neck where surgery had been contra-indicated on cosmetic grounds.

Radiotherapy as an adjuvant to surgery was performed in 5 patients with a 40% 5-year survival. In 4 patients it was given to the primary site alone, while in the fifth it was given prophylactically to the regional nodes as well.

*Inadequate treatment.*—Four cases had gross interference to their primary lesion in the form of diathermy, $CO_2$ snow, silver nitrate, and one lesion was scratched off, before adequate local therapy had been given. Surprisingly, one person survived 5 years.

*Treatment of patients in Stage* 2 (Table VIII)

The number of patients presenting in Stage 2 at the initial examination was 22 and after treatment 4 (18%) survived 5 years.

TABLE VIII.—*Treatment for Stage 2 Melanoma*

| Type of treatment | No. of patients | 5 year survivors |
|---|---|---|
| *Amputation* | | |
| (a) of digit and block dissection | 1 | 0 |
| (b) Hindquarter | 4 | 1 |
| (c) Forequarter | 1 | 0 |
| *Radiotherapy* | | |
| to primary and nodes | 2 | 0 |
| *Local excision* | | |
| (a) with block dissection | 13 | 2 |
| (b) with block dissection and radiotherapy | 1 | 1 |
| Total | 22 | 4 (18%) |

The usual form of therapy in this group was local excision of the primary followed by block dissection of the regional nodes. In 5 cases, major amputative surgery was performed and no patients were treated with radiotherapy alone.

*Treatment and Clinical Features of Recurrent Disease*

Of the 111 patients 77 were later treated for recurrent disease (Table IX). There were 39 males and 38 females. Unless otherwise stated results in this section are based on first recurrence.

TABLE IX.—*Classification of Recurrent Disease According to Site*

| Site | No. of patients | Male | Female |
|---|---|---|---|
| 1. Local recurrence | 21 (27%) | 7 | 14 |
| 2. Local and nodal recurrence | 3 (4%) | — | 3 |
| 3. Nodal recurrence | 32 (42%) | 18 | 14 |
| 4. Widespread recurrence | 21 (27%) | 14 | 7 |
| Total | 77 (100%) | 39 | 38 |

Local recurrences were defined as those occurring at or near the site of primary excision. " In transit " metastasis or satellitosis was included in groups 1 and 2 in Table IX. If there were local recurrences together with evidence of disease elsewhere, apart from the nodes, the disease was classified as being widespread.

It was found that most recurrences occurred within a short time of removal of the primary lesion; 74 % occurred within 1 year, and 88 % within 2 years of removal of the primary. Fig. 2 summarises the relationship between the site of recurrence, its time of onset, and the eventual survival after treatment. Local and nodal recurrences usually occurred within 12 months of treatment of the primary, while widespread disease occurred after an average interval of 25 months.

The commonest site for recurrent disease was in lymph nodes. Some difference between the sexes was apparent at each site but was most marked in the presence of widespread recurrence where males were more frequently affected.

FIG. 2.—Relationship between " free interval " and survival following treatment of recurrence.
" Free interval " ■
Survival following treatment □

When the site of the primary was considered, it was found that lesions of the trunk were less prone to spread via the nodal route, local and widespread disease predominating, but the difference was not significant.

The effect of treatment of primary lesion on the type of recurrent disease is summarised in Table X. When dealing with local recurrences only, there was a

TABLE X.—*Effect of Treatment of the Primary on Subsequent Recurrence (all Episodes)*

| Site of recurrence | Excision | Direct suture | Graft | Not known | Total |
|---|---|---|---|---|---|
| Local | 3 | 19 | 3 | 7 | 32 |
| Nodal | 12 | 22 | 12 | 5 | 51 |
| Widespread | 16 | 29 | 11 | 7 | 63 |
| Not known | 2 | 1 | 2 | 1 | 6 |
| Total recurrences | 33 | 71 | 28 | 20 | 152 |
| Number of patients | 26 | 49 | 28 | 8 | 111 |
| Overall recurrence rate per person | 1·27 | 1·45 | 1·00 | 2·50 | 1·37 |

significantly higher rate of recurrence in patients who had their wounds closed by direct suture rather than by grafting ($\chi^2 = 5{\cdot}6$, $P < 0{\cdot}01$). There seemed to be no relationship between the type of wound closure and the frequency of nodal or widespread recurrence. The inclusion of deep fascia in the excision had no effect.

*Local recurrence.*—The treatment of the 21 patients with locally recurrent disease is summarised in Table XI. It can be seen that surgery took the form of

TABLE XI.—*Treatment of Locally Recurrent Disease and Survival*

| Treatment | No. of patients | 5-year survivors |
|---|---|---|
| Local excision only | 11 | 2 |
| ,, ,, + radiotherapy | 1 | – |
| ,, ,, + chemotherapy | 1 | – |
| Amputation, below knee | 2 | – |
| Radiotherapy alone | 3 | – |
| No treatment or unknown treatment | 3 | 1 |
| Total | 21 | 3 (14%) |

local excision or amputation. Six had further local recurrence, 7 had nodal recurrence, and 8 (including the 3 untreated cases) died of widespread disease. Therefore 13 patients out of 21 were suitable for futher surgical treatment.

*Local and nodal recurrence.*—The 3 patients (Table IX) were treated as follows: 1 had local removal followed by block dissection; 2 had radical surgery, 1 a hind-quarter, and the other a forequarter, and both are alive and well 15 years later!

*Nodal recurrence.*—Table XII summarises the treatment given to the 32 patients. The survival in patients who have their nodes dissected only when they become clinically enlarged is low, only 12 % survived for 5 years. A further follow-up of these 32 patients after treatment showed that the next recurrence took the form of widespread disease in 26. In only 6 patients (2 with local, and 4 with nodal) was further surgery possible.

*Widespread recurrence.*—Chemotherapy was mainly given in the form of nitrogen mustard, and one patient had her pituitary removed. No form of treatment seemed to be of use at this stage.

TABLE XII.—*Treatment of Nodal Recurrence and Survival*

| Treatment | No. of patients | 5-year survivors |
|---|---|---|
| Block dissectiom | 21 | 3 |
| ,, ,, + radiotherapy | 2 | – |
| ,, ,, + cytotoxic agents | 2 | – |
| Hindquarter amputation | 2 | 1 |
| Radiotherapy alone | 2 | – |
| Unknown | 3 | – |
| Total | 32 | 4 (12%) |

Two other aspects of recurrent disease need to be discussed, namely satellitosis and major amputation.

*Satellitosis.*—This was defined as secondary deposits occurring in the lymphatic drainage of a primary lesion, and due to lymph stasis, the latter being due to either deposits of tumour in lymph nodes or to removal of regional nodes. There were only 7 patients in this series, an incidence of 6 %. The nodes were clinically involved in 4, and the other 3 became positive on follow-up. Satellitosis, it is said, is always a danger when positive nodes are removed, but in our series only 2 out of 32 developed this complication. When the nodes were involved the patients were far more likely to die of widespread disease.

*Major amputation.*—Major amputative surgery in this disease (Table XIII) gives survival rates which compare favourably with that for the whole series. The number of cases is too small to assess the value of this form of surgery.

TABLE XIII.—*Survival after Various Types of Major Amputative Surgery*

| Stage of disease | Type of amputation | No. of patients | 5-year survivors |
|---|---|---|---|
| *Primary* | | | |
| Stage 1 | below knee | 2 | 1 |
| Stage 2 | hindquarter (4)<br>forequarter (1) | 5 | 1 |
| *1st Recurrence* | | | |
| Local | below knee | 2 | – |
| Local and Nodal | hindquarter<br>forequarter | 2 | 2 |
| Nodal | hindquarter | 2 | 1 |
| *2nd Recurrence* | | | |
| Nodal | hindquarter | 1 | – |
| Total | | 14 | 5 (36%) |

### DISCUSSION

Our overall survival rate of 34 % is comparable with recent reports, 41 %, Wright *et al.* (1953), 39·1 %, Pack (1959), 40 %, Cade (1961), 32 %, James (1961), 40 %, Watson (1964). Earlier reports showed a poorer prognosis, 11·1 %, Affleck (1936), 18·3 % Daland and Holmes (1939) and 9·7 % Raven (1950). In our series extending from 1941–61 we found no significant improvement in survival rates over these years.

Cade (1961) and Watson (1964) stress that due to the frequency of late recurrences survival analysis should be based on follow up periods greater than 5 years. We agree with Wright *et al.* (1953) and Vogler *et al.* (1958) that the majority of recurrences occur in the first 5 years (88 % in 2 years in our series), which justifies our usage of 5 years survival data, and that the 2-year survival rate—which includes 88 % of the recurrences—would be a valuable index of the efficiency of a method of treatment.

Malignant melanoma has never been classified as an endocrine-dependant tumour (Huggins, 1965) though there are some features to suggest that its biological activity may be related to the endocrine system. We found a 1·3 : 1 female to male ratio which, in this small sample, varied considerably within age groups. Nitter (1956) reported a high incidence in menopausal patients with associated low survival, but the survival rate of our patients in the age range 45–54 was not markedly lower than that in other age groups. It is universally accepted in melanoma that survival rates are significantly higher in the female, though White (1959) has stressed that a survival over 5 years is needed before this becomes apparent. In our series the difference in survivorship between females and males became apparent during the first 5 years, and at 5 years, though not statistically significant was most pronounced, and then remained constant (Fig. 1).

We were unable to show any statistical difference in survival rates between the age decades with the exception of older males, who had a decreased survival rate. Block and Hartwell (1961) have shown that in contrast to other tumours there is decreasing survival with age. Wright *et al.* (1953) emphasised the high survival rate of young female patients.

The size of the lesion has a profound effect on prognosis, only 9 % of patients with lesions larger than 3 cm. surviving 5 years (compared to 49 % with lesions smaller than 3 cm.). Lesions of comparable size seemed to carry a better prognosis when situated on the lower limb. Pack *et al.* (1952) have attributed this to the uniform lymphatic drainage of this site. The lower limb, and head and neck lesions have a good prognosis and the predominance of lesions at these sites amongst younger and older women respectively, might account for the failure of age to decrease survival in the female. Lesions of the trunk carry a poor prognosis which has been attributed to its diffuse lymphatic drainage.

Mundth *et al.* (1965) showed that with equal duration of symptoms there was a much higher percentage of males presenting with enlarged regional nodes. This suggests that the disease is more active in the male. This could not be demonstrated though there were certain features to suggest that the disease was biologically less active in the female, viz:

(a) They presented with smaller lesions.
(b) When the melanoma arose from a pre-existing lesion the survival rate was greater for females than for males.
(c) The difference in survival between patients with palpable and impalpable nodes was less marked in the female.

Lea (1965) has emphasised the role of trauma as an aetiological factor. We felt like others (Catlin, 1954; Cade, 1961) that it only served to draw attention to a lesion already undergoing malignant change.

We found that incisional biopsy and gross interference with the primary lesion did not appreciably alter the survival or recurrence rate. This is probably accounted for by the small numbers in the series, and we felt that any interference is best avoided.

We would advocate that the primary should be treated by wide removal of the skin and subcutaneous tissue followed by split skin grafting. Following such treatment we found that the incidence of local recurrence is significantly reduced. Our results show that removal of deep fascia had no effect on survival. Olsen (1966) found that removal of deep fascia increases the spread to regional nodes by destroying a system of lymphatic valves, and he showed by lymphangiographic techniques that these valves direct lymph superficially from the deep fascia, and not towards it as was thought previously (Handley, 1907).

The value of radiotherapy has been underestimated, and Cade (1961) has drawn attention to the work of Ellis (1939) who successfully treated 38 patients with D.X.R. alone. Two of 4 of our patients treated only by D.X.R. survived 5 years. Nitter (1956) and Dickson (1958) have shown that the results from surgery alone can be improved by prophylactic, post-operative radiotherapy to the operative site and/or to the regional nodes. In our 5 patients treated in this way, 2 survived 5 years. We would therefore disagree with the statement that " Malignant melanoma is as resistant as the surrounding skin " (Wright *et al.*, 1953).

Even though Handley in 1907 wrote " the problem is not the excision of an organ, but the extirpation of a diseased lymphatic area ", we are still doubtful how far to extend surgical excision. In only 2 cases was prophylactic nodal dissection performed, both survived for 5 years and had histologically negative nodes. In such cases with clinically negative nodes 20–50 % will be found to have histological involvement (Fortner *et al.*, 1964).

The low 12 % 5-year survival rate in this series following therapeutic nodal dissection is fairly representative (Fortner *et al.*, 1964). It suggests that the removal of nodes before they become involved would improve survival. Spread to regional nodes is the commonest form of metastasis in this series. Recent anatomical studies by Pressman and Simon (1961), show that direct communications exist between lymph nodes and the venous system, allowing the passage of fluid, cells and air. This would explain our finding of over 80 % of patients with recurrent nodal disease dying of widespread involvement within a short period of time, and would indicate that the disease is at an advanced stage when regional nodes become involved. Removal of these nodes at an early stage might therefore remove an important pathway of spread. However, despite the overwhelming evidence for prophylactic nodal dissection in some series (Mundth *et al.*, 1965) others disagree (Sandeman, 1966; McNeer and Das Gupta, 1964), and the case still remains to be proven.

In many cancers, e.g. carcinoma of the breast, the length of the so-called " free interval " between treatment and recurrence is one of the most important clinical indications in assessing further survival. In cases of malignant melanoma with recurrence we found, to our surprise, that the shortest free interval was associated with the best prognosis and this occurred in locally recurrent disease (Fig. 2). The long " free-interval ", and then the rapid lethal effect of widespread involvement is similar to the classical description in ocular melanoma by Wilbur and Hartman (1931). The length of the " free-interval " for nodal recurrent disease falls between that for local and widespread recurrent disease.

The average length of survival in each type of recurrent disease was approximately the same (Fig. 2) and would suggest that the biological activity of this tumour may be pre-determined and that consequently treatment may have little effect on the eventual outcome. Yet we were impressed by the value of radical surgery at all stages of the disease, and particularly the value of major amputative surgery (Table XIII). McPeak *et al.* (1963) gave a survival rate of 30 % for major amputative surgery which is similar to our figure of 36 %.

### SUMMARY

A retrospective study of 111 patients with malignant melanoma of the skin, seen in South Wales between the years 1941–1961 is described. The overall 5-year survival rate was 34 %.

1. The series included a slight excess of females and though not significant, the survival rates were generally higher in females.

2. Lesions occurred most frequently in the lower limb followed by head and neck site, trunk, and upper limb. The order was subject to reversal by the influence of age and sex. Lesions of the lower limb were most frequent among younger females, head and neck among older females and trunk among younger males.

3. At the lower limb the average age of occurrence in females was 45 years and in males 12 years later. This low average age for females also contrasted with the age at which they were affected at other sites—an average of 9½ years later.

4. Lesions of the lower limb carried the best prognosis for both males and females, followed by head and neck lesions, upper limb and trunk. Lesions of the trunk resulted in a low rate of survival particularly among males and a further vulnerable group was that of older males, irrespective of site of lesion.

5. In females the disease presented as smaller lesions than in males. Small lesions ($< 3$ cm. in diameter) in both sexes showed a longer survival period than larger lesions ($> 3$ cm. in diameter).

6. In both sexes, the incidence of nodal metastasis was similar and decreased the survival rate. The effect was less marked in females.

7. The duration of symptoms was directly related to survival, long duration was associated with better prognosis.

8. Treatment: the best survival followed wide local excision of the primary lesion, surrounding skin and subcutaneous tissue. The removal of deep fascia did not affect survival. A uniformly low survival rate followed therapeutic nodal dissection. Our evidence suggests that prophylactic nodal dissection might well improve prognosis.

9. Radiotherapy alone is of some value in a few selected cases of head and neck lesions. It was found impossible to assess the value of radiotherapy, ablative hormone therapy and chemotherapy as adjuvants to surgery.

10. 88 % of recurrent disease occurred within the first 2 years following treatment. Five-year survival rates were therefore of value in assessing prognosis.

11. The length of the so-called "free-interval" was found to be inversely related to survival time and of little value on its own in assessing prognosis.

### References

Affleck, D. H.—(1936) *Am. J. Cancer*, **27**, 120.
Beerman, H., Lane, R. A. G. and Shaffer, B.—(1955) *Am. J. med. Sci.*, **229**, 583.
Block, G. E. and Hartwell, S. W.—(1961) *Ann. Surg.*, **154**, 74.
Cade, S.—(1961) *Ann. R. Coll. Surg.*, **28**, 331.
Catlin, D.—(1954) *Ann. Surg.*, **140**, 796.
Daland, E. M. and Holmes, J. A.—(1939) *New Engl. J. Med.*, **220**, 651.

Davis, N. C. and Herron, J. J. F.—(1966) *Med. J. Aust.*, **1**, 643.
Dickson, R. J.—(1958) *Am. J. Roentg.*, **79**, 1063.
Ellis, F.—(1939) *Br. J. Radiol.*, **12**, 327.
Fortner, J. G., Booher, R. J. and Pack, G. T.—(1964) *Surgery, St. Louis*, **55**, 485.
Handley, W. S.—(1907) *Lancet*, i, 927 and 996.
Huggins, C. B.—(1965) *J. Am. med. Ass.*, **192**, 1141.
James, A. G.—(1961) *J. Am. med. Ass.*, **176**, 5.
Lea, A. J.—(1965) *Ann. R. Coll. Surg.*, **37**, 169.
McNeer, G. and Das Gupta, T.—(1964) *Surgery St. Louis*, **56**, 512.
McPeak, C. J., McNeer, G. P., Whiteley, H. W. and Booher, R. J.—(1963) *Surgery St. Louis*, **54**, 426.
Mundth, E. D., Guralnick, E. A. and Raker, J. W.—(1965) *Ann. Surg.*, **162**, 15.
Nitter, L.—(1956) *Acta radiol.*, **46**, 547.
Olsen, G.—(1966) *Acta chir. scand; Suppl.*, **365**, 1.
Pack, G. T.—(1959) *Surgery St. Louis.*, **46**, 447.
Pack, G. T., Gerber, D. M. and Scharnagel, I.—(1952) *Ann. Surg.*, **136**, 905.
Pressman, J. J. and Simon, M. B.—(1961) *Surgery Gynec. Obstet.*, **113**, 537.
Raven, R. W.—(1950) *Ann. R. Coll. Surg.*, **6**, 28.
Sandeman, T. F.—(1966) *Lancet*, i, 602.
Urtega, O. and Pack, G. T.—(1966) *Cancer, N.Y.*, **19**, 607.
Vogler, W. R., Perdue, G. D. and Wilkins, S. A.—(1958) *Surgery Gynec. Obstet.*, **106**, 586.
Watson, E. C.—(1964) *Aust. N.Z. J. Surg.*, **33**, 31.
White, L. P.—(1959) *New Engl. J. Med.*, **260**, 789.
Wilbur, D. L. and Hartman, H. R.—(1931) *Ann. intern. Med.*, **5**, 201.
Williams, W. J., Davies, K., Jones, W. M. and Roberts, M. M.—(1968) *Br. J. Cancer*, **22**, 452.
Wright, R. B., Clark, D. H. and Milne, J. A.—(1953) *Br. J. Surg.*, **40**, 360.